# Segmented optimization for river ecological corridor width based on ecosystem services: a case study of the North Canal River, China

**DOI:** 10.1038/s41598-026-43293-2

**Published:** 2026-03-22

**Authors:** Liying Zhu, Yuansong Wei, Jie Zhao, Dawei Yu, Wenjing Zhang, Min Yan, Xinyuan Xu

**Affiliations:** 1 State Key Laboratory of Regional Environment and Sustainability, Research Center for Eco–Environmental Sciences, Chinese Academy of Sciences, Beijing, 100085 China; 2 Laboratory of Water Pollution Control Technology, Research Center for Eco–Environmental Sciences, Chinese Academy of Sciences, Beijing, 100085 China; 3 University of Chinese Academy of Sciences, Beijing, 100049 China; 4 Beijing Municipal Institute of City Planning & Design, Beijing, 100045 China; 5 Ministry of Ecology and Environment, Chinese Academy of Environmental Planning, Beijing, 100041 China; 6 Key Laboratory of Digital Earth Science, Aerospace Information Research Institute, Chinese Academy of Sciences, Beijing, 100094 China

**Keywords:** Ecological corridors, Ecosystem services, Width threshold, Segmented regression, urban–rural gradient, North Canal River, Ecology, Ecology, Environmental sciences, Environmental social sciences

## Abstract

Adapting to climate change requires a better understanding of how the spatial configuration of river ecological corridors influences both ecological connectivity and ecosystem service delivery. Corridor width is a critical factor in corridor planning, yet uniform design approaches often overlook the strong spatial heterogeneity between urban and rural river segments. In this study, we investigated the North Canal River in Beijing, quantifying the effects of corridor width on ecosystem service value through integration land use data (1990–2020), landscape pattern metrics, and segmented regression analysis. The results revealed pronounced contrasts in land–use trajectories between urban and rural corridors. Urban cropland declined by more than 70%, while built–up land expanded substantially before partially retreating following large–scale ecological restoration after 2015. Rural corridors experienced more moderate cropland loss and consistently maintained higher landscape aggregation and lower fragmentation. Increasing corridor width enhanced spatial connectivity and landscape stability, particularly in rural segments. Ecosystem service value exhibited a clear non–linear response to corridor width. Urban river corridors were increasingly dominated by cultural services, whereas rural corridors retained robust regulation and supporting services. Breakpoint models identified service–based width thresholds at approximately 126 m for urban and 311 m for rural corridors, respectively. These findings underscore the importance of context–specific corridor design that accounts for spatial heterogeneity in ecosystem service responses and provide quantitative support for adaptive river corridor planning in rapidly urbanizing regions.

**Supplementary Information:**

The online version contains supplementary material available at 10.1038/s41598-026-43293-2.

## Introduction

Urban river ecological corridors are increasingly recognized as critical resilience–building components of urban ecological infrastructure, playing essential roles in mitigating climate–induced risks, sustaining ecosystem services (ESs), and enhancing urban adapative capacity^1, 2, 55^ UNEP, 2023). Globally, as cities expand and human pressures intensify, these corridors frequently undergo fragmentation, channelization, or encroachment, undermining their ability to sustain ecological functions^3, 4^. Meanwhile, in many rapidly urbanizing regions–especially in East and South Asia, and parts of Africa and Latin America–riparian corridors face profound land–use changes and infrastructural expansion, which compromise their multifunctional roles. In contrast, initiatives across Europe and North America increasingly reposition river corridors as critical elements of blue–green infrastructure for flood mitigation, habitat connectivity, and urban livability^5, 6^. These contrasting trajectories underscore a critical insight: river corridors are not uniform entities but exhibit strong spatial heterogeneity along urban–rural gradients shaped by varying levels of socioeconomic development. Recognizing this heterogeneity is essential to move beyond one–size–fits–all designs toward context–sensitive corridor planning that aligns with localized ecological functions and societal demands. Despite their importance, defining the appropriate spatial width of urban river ecological corridors in practice remains highly challenging because ecological functions, land–use constraints, and service priorities vary sharply across segments, which often leads to inconsistent or arbitrary width standards across regions.

In rapidly urbanizing megacities such as Beijing, river corridors serve as vital ecological networks that interconnect fragmented blue–green spaces within metropolitan landscapes. The North Canal River, the largest tributary of the Hai River basin, exemplifies such complexity by displaying pronounced spatial heterogeneity along its urban–rural continuum. Rural segments predominantly support water conservation, soil retention, and biodiversity maintenance, whereas urban segments are oriented toward recreational, aesthetic, and cultural services^7, 8^. These functional differences are accompanied by distinct pressures: urban areas face land–use intensification and habitat fragmentation, while rural areas are more affected by water depletion and agricultural expansion^9^. This divergence raises a critical planning question: under land–use constraints, how can urban and rural river corridors be spatially differentiated to optimize ecosystem service benefits? Addressing this question is increasingly urgent as cities seek nature–based solutions (NBSs) to balance ecological restoration, climate resilience, and urban development.

The dynamic interplay between ecological processes and human activities has long been a central focus in coupled human–natural systems theory^10^. Within this theoretical framework, landscape ecological research has evolved from describing spatial patterns to understanding how pattern influences process and, in turn, how process shapes service outcomes. Fu,^11^ further extended this understanding through the “pattern–process–service–sustainability” paradigm, emphasizing that sustainable landscape management requires elucidating the mechanisms linking spatial configuration, ecosystem functioning, and service delivery. Applying this paradigm to urban river corridors provides a theoretical lens for analyzing how spatial form, especially corridor width, mediates ecosystem service provision under varying levels of human disturbance.

Despite these theoretical advances, most studies of ecological corridors and ecosystem services have approached the topic from separate perspectives. Corridor planning research has typically focused on connectivity, structural metrics, or least–cost path analyses^12, 13, 14^, yet few have calibrated corridor width in relation to ESs performance. On the other hand, ecosystem service studies have substantially advanced understanding of non–linear behaviors and threshold effects. For instance, Yan et al.,^15^ identified response patterns of ESs to landscape gradients; Zhu et al.^16^, examined critical thresholds linking ESs and human well–being; Zhang et al., ;^17^ explored the non–linear impacts of socio–ecological drivers on service dynamics; and Yao et al., and^18^ evaluated the threshold effects of human activities on urban ES values. However, there is still little knowledge about the marginal influence of spatial configuration (such as corridor width) on the capacity of riverine landscapes to deliver multiple ESs, which is essential for developing precision–based ecological corridor design and management strategies at regional scales. Systematic reviews of urban river ESs highlight that while regulating services dominate past studies, cultural services remain underexplored and spatial configuration effects are inadequately linked to design^3^. The concept of “proactive corridor definition” suggests corridors be defined not by arbitrary widths but by the functional space needed to sustain key river processes^6^. Mapping functional connectivity across services offers methodological inspiration but has not translated into corridor width prescriptions^5^.

More international research recognizes the need to account for scale dependence, trade–offs, and threshold effects in spatial design. In Europe, adaptive corridor and riparian buffer designs are increasingly informed by floodplain hydrology and habitat–sensitivity assessments, resulting in spatially variable buffer widths that target nutrient retention and biodiversity outcomes^19^, whereas in the United States, riparian buffers are defined according to stream order and watershed characteristics^20^. In developing countries, large–scale river rehabilitation programs, such as China’s Ecological Redline Initiative and India’s National River Conservation Plan, aim to restore multifunctionality^21, 22^. However, explicit empirical identification of width thresholds for maximizing aggregated service value across segments is rare. Some recent advances in threshold detection and segmented regression^15, 16^ and socio–ecological driver analyses^17^ suggest the feasibility of such quantification.

In this study, we address this research gap by combining multi–decadal land–use data (1990–2020), landscape pattern metrics, and ecosystem service valuation to explore how river ecological corridor width influences ES performance along the urban–rural gradient of the North Canal River in Beijing. Using segmented regression analysis, we identify the width ranges where ESV increases most efficiently and where expansion yields diminishing ecological benefits. Grounded on the pattern–process–service–sustainability paradigm, this study tests two hypotheses as follows: (1) driven by distinct land–use transitions and landscape configurations, functional divergence between urban and rural segments results in markedly different ES profiles; and (2) ESV exhibits segment–specific nonlinear responses to corridor width, with distinct optimal thresholds reflecting divergent service priorities. Overall, the empirical results reveal how the functional differentiation between urban and rural river segments drives variations in ecosystem service performance and identify the optimal corridor widths for urban and rural sections that most effectively enhance ecological benefits. By integrating these findings, this study provides a service–based, precision corridor design framework for river systems worldwide facing spatial heterogeneity and competing land–use pressures.

## Materials

### Study area

**Fig. 1:**
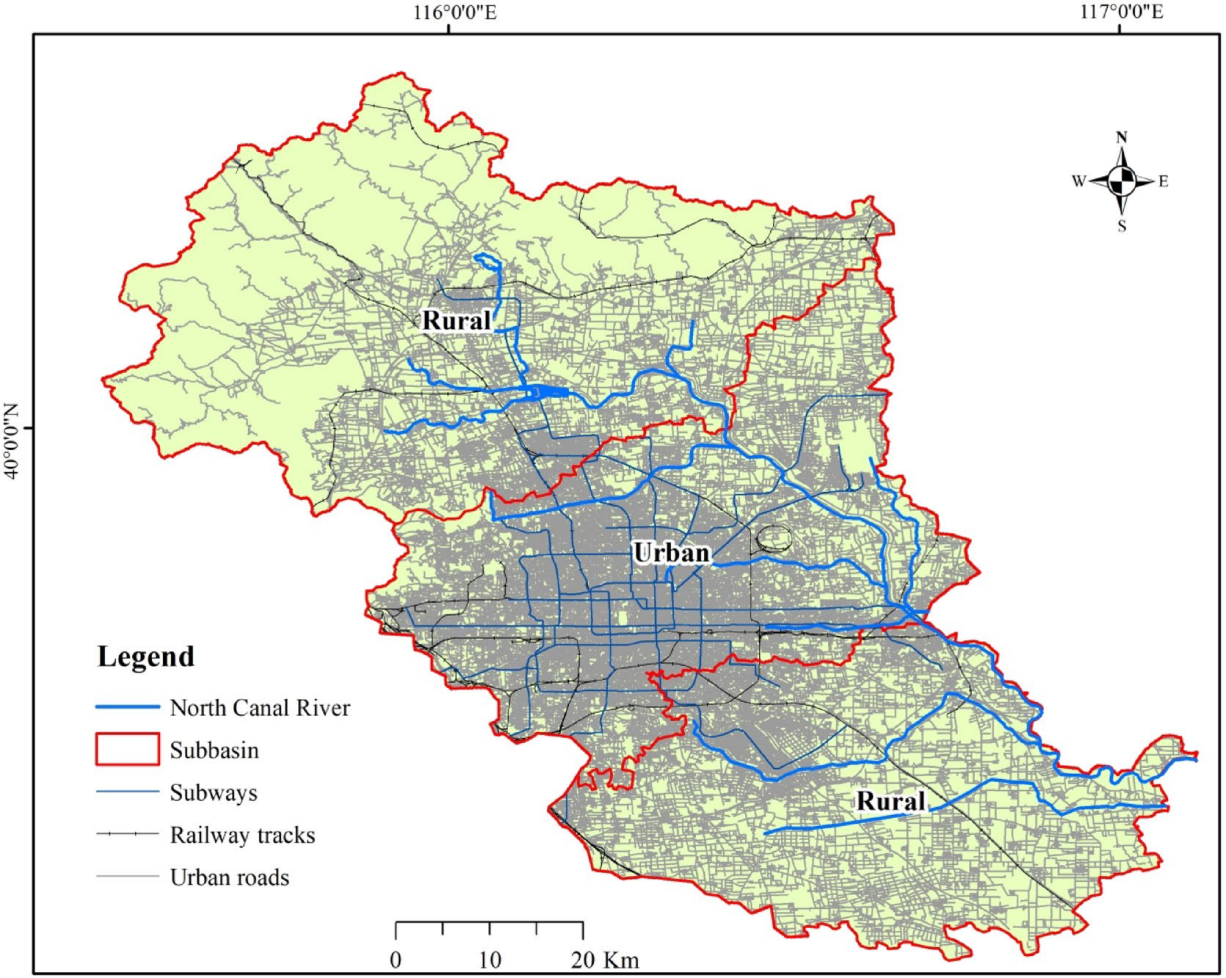
Urban development levels in the North Canal River basin.

The North Canal River, a principal tributary of the Haihe River system, traverses the eastern suburban areas of Beijing and Tianjin. The study area encompasses the Beijing section of the river, which provides multiple key ecosystem services. It functions as a critical corridor for flood regulation and drainage in the metropolitan area, while also maintaining hydrological connectivity between the central urban zone and the sub–administrative center through its integrated blue–green infrastructure. Designated as a core component of the North Canal Green Ecological Corridor under both The Master Plan of Development for Beijing (2016–2035) and Beijing Garden City Special Planning (2023–2035), this area provides critical regulating services, e.g., microclimate moderation and habitat provisioning. In addition, it delivers cultural services reflecting the city’s rich historical heritage, with these complementary ecosystem services collectively underpinning regional biodiversity conservation and improving city livability. The basin spans eight administrative districts across a northwest–southeast transect, covering a total area of 4,348 km² with a main channel length of 89.4 km. Key biophysical drivers influencing ecosystem service provision include the region’s temperate monsoon climate, characterized by a mean annual precipitation of 643 mm, with pronounced seasonal variability, approximately 84% of rainfall occurs during the June–September rainy season, while October through May constitutes the dry period^23^. In terms of spatial structure, urbanized land uses are predominantly concentrated in the mid–reaches of the watershed, gradually decreasing toward the upstream and downstream sections, forming a distinct rural–urban–rural gradient (Fig. 1).

### Land use types of data

The 1990, 1995, 2000, 2005, 2010, 2015 and 2020 land use data of the North Canal River Basin were sourced from the Resource and Environment Data Center of the Chinese Academy of Sciences (https://www.resdc.cn/). By supervised classification of Landsat–TM images, the LULC of the study area was classified into six main types of Croplands, Forestland, Grassland, Water, Built–up land, and Unused land, respectively. After verification of random sampling at field investigation points and Kappa coefficient accuracy assessment, the accuracy of LULC classification results in all seven phases was higher than 85%^24^. The watershed was divided into sub–regions by integrating the administrative divisions of Beijing’s urban districts with the segment boundaries of the Wenyu River, specifically Shazi Ying and Beiguan Gate. The section from Shazi Ying to Beiguan Gate was designated as the urban segment, primarily located within the main urban area, while the remaining sections were classified as rural segments.

### Corridor width

Based on the distinct land–use patterns between urban and rural river segments, we defined separate corridor width gradients to reflect their differing ecological functions and spatial contexts. Urban corridors require widths that are sensitive to ecosystem services responses under intense land–use constraints, whereas rural corridors require broader widths to accommodate larger functional spaces. Accordingly, urban corridor widths were set at 50, 100, 150, 200, 250, and 300 m, while rural corridor widths were set at 100, 200, 300, 400, 500, and 600 m.

The selection of these width ranges was informed by river management practice, the spatial characteristics of the North Canal River basin, and empirical literature on riparian corridors and functional river space. Extensive research indicates that key riparian ecological functions, including nutrient retention, habitat provision, and hydrological regulation, are most sensitive to buffer widths within the first tens to hundreds of meters from the channel, with ecological responses generally diminishing beyond this range^6, 25, 20^. In urban areas, corridor and riparian buffer widths are typically constrained to 30–200 m, reflecting physical limitations imposed by dense built–up areas and flood control infrastructure^22, 20^. In contrast, rural river corridors often retain more extensive floodplains, where lateral hydrological connectivity, sediment exchange, and habitat mosaics remain active, resulting in functional river spaces that frequently span several hundred meters (O’Briain, 2019;^54,^ ^19, 4^. The North Canal River was originally constructed in 1205 as an artificial waterway, developed by dredging and modifying a natural river to support urban waterborne transportation^26^. Consequently, its catchment area is relatively narrow. As shown in Fig. 1, the buffer width along the main channel in the downstream is limited. Therefore, the rural corridor width was extended up to 600 m, which sufficiently encompasses the potential floodplain process zones and ecological buffering capacity.

Furthermore, this multi–level gradient design ensures sufficient data density to reliably identify response relationships between corridor width and ecosystem service value, providing a robust foundation for the segmented regression analysis applied in this study.

## Methodology

### Landscape pattern

Based on the hierarchical framework of landscape system theory^27^, landscape metrics can be classified into three distinct levels: patch, class, and landscape. In accordance with this conceptual structure, this study selected a set of six representative and widely adopted landscape–level indices using Fragstats 4.2.1 software (developed by Oregon State University), as summarized in Table 1. The chosen metrics include Number of Patches (NP), Patch Density (PD), Largest Patch Index (LPI), Landscape Shape Index (LSI), Contagion Index (CONTAG), and Shannon’s Diversity Index (SHDI). These indicators collectively capture essential spatial and structural characteristics of the landscape, enabling a multidimensional analysis of pattern complexity, fragmentation, dominance, and diversity. By integrating metrics that reflect both compositional and configurational attributes, this selection provides a robust basis for quantifying and interpreting landscape changes in a systematic manner.

**Table 1:** The value coefficient of ecosystem services on six land use categories in the North Canal River basin. (CNY/hm^2^/yr).

Ecosystem classification	Cropland	Grassland	Forestland	Water	Built-upland	Unused land
Provision services						
FP	1102.48	492.87	327.50	1037.62	0.00	0.00
RM	518.81	726.34	752.28	298.32	0.00	0.00
WS	25.94	402.08	389.11	10752.38	0.00	0.00
Regulation services						
GR	869.01	2555.15	2474.08	998.71	0.00	25.94
CR	466.93	6757.53	7402.80	2970.20	0.00	0.00
WT	129.70	2230.89	2169.28	7198.52	0.00	129.70
WR	350.20	4954.65	4844.41	132608.34	0.00	38.91
Supporting Services						
EP	1335.94	3112.87	3012.35	1206.24	0.00	25.94
MSF	155.64	233.47	230.22	90.79	0.00	0.00
Hab	168.61	2827.53	2743.22	3307.43	0.00	25.94
Cultural services						
Cul	77.82	1245.15	1203.00	2451.39	12230.10	12.97

FP – Food provision, RM – Raw materials, WS – Water supply, GR – Gas Regulation, CR – Climate Regulation, WT – Waste Treatment, WR – Water Regulation, EP – Erosion Prevention, MSF – Maintenance of Soil Fertility, Hab – Habitat, Cul - Cultural.

### Assessment of ESV

#### Regional calibration of equivalence coefficients

To improve the regional representativeness of ecosystem service valuation, the equivalence factor was not directly adopted from literature values but calibrated using long–term Chinese agricultural statistics. Following Xie et al.,^28^, the national net profit from grain production per unit area was used as the baseline for standard equivalence factors, while multi–year data for rice, wheat, and maize were incorporated to reflect long–term productivity and socio–economic conditions. The formula is as follows:

$$D={S}_{r}\times{F}_{r}+{S}_{w}\times{F}_{w}+{S}_{c}\times{F}_{c}$$

Where *D* represents 1 standard equivalence factor for the value of *ES*_*s*_ (CNY/ha); *Sr*, *S*_*w*_ and *S*_*c*_ represent the percentage (%) of area sown with rice, wheat and corn, respectively, to the total area sown with the three crops in that year; *F*_*r*_, *F*_*w*_ and *F*_*c*_ represent the national average net profit per unit area for rice, wheat and corn in that year (CNY/ha), respectively. The average net profit per unit area and sown area of rice, wheat, and corn were collected from the “China Statistical Yearbook 1990–2021” and “National Agricultural Product Cost and Profit Data Compilation 1987–2021”. To reduce the influence of short–term market fluctuations and inflation, average net profits over multiple years were used to derive a stable regional equivalence coefficient. This calibration ensured that the equivalence factor reflected long–term agricultural output and local economic context rather than annual price volatility. The resulting value (~ 1297 CNY/ha·yr) was subsequently applied to localize the ecosystem service coefficients proposed by Xie et al.,^29^ for the North Canal River Basin.

#### Zonal Adjustment of Value Coefficients

Based on the regionally calibrated equivalence factor, unit ecosystem service value coefficients (CNY/ha/year) for different land–use types were derived by multiplying the equivalence factor with the ecosystem service coefficients proposed by Xie et al.,^29^. These coefficients formed the baseline valuation system for cropland, forestland, grassland, water bodies, and unused land.

Based on the corrected coefficient and the standard value of equivalent factor for ecological services in the Basin, the unit ecosystem value coefficient (CNY/ha/year) can be gained, and the calculation formula was as follows:

1 $${\mathrm{V}\mathrm{C}}_{fi}=D\times{\mathrm{E}\mathrm{C}}_{fi}$$

Where *VC*_*fi*_ is the value of ecosystem service *f* of land use type *i* (CNY/ha); and *EC*_*fi*_ is the equivalent coefficients of ecosystem service *f* of land use type *i* suggested by Xie et al.,2017^29^.

Given the distinct ecosystem functions and socio–environmental interactions between rural and urban corridors, zone–specific value coefficient (VC) systems were developed rather than applying a uniform valuation scheme. For cropland, forestland, grassland, water bodies, and unused land, the value coefficients were derived by combining the regionally calibrated equivalence factor with the ecosystem service coefficients proposed by Xie et al.,^29^. This ensured consistency with widely used national–scale valuation frameworks, while maintaining regional economic relevance through local calibration.

Built–up land has traditionally been excluded from ecosystem service value assessments due to its relatively low biophysical service provisioning capacity and thus minimal estimated ecological value^30^. However, within highly urbanized river corridors, built–up areas integrated into blue–green infrastructure systems increasingly contribute to cultural ecosystem services such as recreation, aesthetic appreciation, and environmental education^31, 32^. To account for these non–material benefits, this study employed a market–based proxy method to incorporate cultural services into the valuation of built–up land, assigning it a cultural ecosystem service value coefficient for the urban corridor.

Annual tourism and leisure revenue data associated with the North Canal River were collected from the Beijing Statistical Yearbook, Water Resources Bulletins, and Beijing Cultural and Tourism Statistical Bulletins for the period 1990–2020. For each year, the total river–related tourism revenue at the basin scale was spatially allocated to built–up land within the defined urban corridor buffers using an area–weighted approach. To reduce the influence of short–term economic fluctuations, the multi–year average of the calculated coefficients was adopted as the representative cultural service value coefficient for built–up land. Specifically, the coefficient was derived by dividing the annual basin–wide river–related total tourism revenue by the total area of built–up land within the corresponding corridor, resulting in a value of 12230.1 CNY/hm^2^·yr.

To ensure comparability of cultural service values across different corridor width scenarios, the same annual tourism revenue dataset was applied to all width configurations, while the area of built–up land was adjusted according to the specific corridor width. This design enables the cultural ecosystem service value to respond dynamically to land–use changes and corridor expansion. The method captures actual leisure and aesthetic benefits as reflected through market transactions. Although it does not explicitly account for factors such as tourism origin, travel cost, or consumer surplus, income–based proxy indicators have been widely utilized in large–scale ecosystem service assessments to approximate cultural service value^31, 33, 32^. Additionally, a sensitivity analysis of the ecosystem value coefficients was performed to evaluate how uncertainty in these coefficients affects the robustness of the overall valuation results. The complete set of ecosystem service coefficients for land use types within the basin is presented in Table 1.

The total ecosystem service values of different ecological corridors were calculated using the area of land use types such as cropland, grasslands, forestland, water, Built–upland and unused land in the study area. The ecosystem services in four categories devised from the ecosystem services list by Xie et al.,^29^ were calculated as follows:

2 $${\mathrm{E}\mathrm{S}\mathrm{V}}_{f}=\sum_{i=1}^{n}({A}_{i}\times{\mathrm{V}\mathrm{C}}_{fi})$$

Where ESV_*f*_ is the ecosystem value of service *f* (CNY); *A*_*i*_ is the area of land use type *i* (ha); and VC_*fi*_ is the value of ecosystem service f of land use type *i* (CNY /ha).

### Sensitivity analysis of ESV in the North Canal River basin

To assess the robustness of ESV estimation and evaluate their dependence on valuation assumption, a sensitivity analysis was performed based on the coefficient of sensitivity (CS) approach, as proposed by Kreuter et al.,^33^. This analysis tests the responsiveness of total ESV to variations in the assigned value coefficients (VCs) under the zone–specific CV system, which accounts for spatial heterogeneity in ecosystem functions and land use types. The CS is calculated as:

3 $$\mathrm{C}\mathrm{S}=\left|\frac{({\mathrm{E}\mathrm{S}\mathrm{V}}_{j}-{\mathrm{E}\mathrm{S}\mathrm{V}}_{i})/{\mathrm{E}\mathrm{S}\mathrm{V}}_{i}}{({\mathrm{V}\mathrm{C}}_{jk}-{\mathrm{V}\mathrm{C}}_{ik})/{\mathrm{V}\mathrm{C}}_{ik}}\right|$$

where CS is the sensitivity of coefficient; ESV_*i*_ and ESV_*j*_ are the ecological service values before and after adjustment, respectively; VC_*ik*_ and VC_*jk*_ are the coefficients of ecological service values per unit area of the ecosystem *k* before and after adjustment, respectively. If CS > 1, indicating that ESV is elastic with respect to VC; thus, when CS < 1, indicating that ESV is inelastic with respect to VC, and VC is applicable to the estimation of ESV in the study area, even if the accuracy of VC values used as proxy estimation is low, the results of estimation of ESV are credible^33, 34^.

### Determination of the optimal corridor width

Breakpoint analysis was employed to determine the optimal widths of rural and urban corridors independently. This analysis was conducted using the Python library *pwlf* (piecewise linear fitting), which estimates piecewise linear relationships in one–dimensional datasets. In this approach, breakpoints denote the transition points between fitted linear segments^35, 36^; Jekel & Venter, 2019^37^). Breakpoint analysis has been extensively applied across disciplines, particularly in environmental sciences and medicine. For instance, Ollerton et al.,^38^ analyzed historical records to detect shifts in extinction rates of bee and flower–visiting wasp species in Britain, identifying breakpoints in 1928, 1958, 1974, and 1986. Similarly, Villarini et al.,^39^ applied segmented regression to assess abrupt changes and temporal trends in the frequency of heavy rainfall events across the central United States. In a different context, Jauk et al.,^40^ employed segmented linear regression to examine breakpoint effects in Intelligence data, thereby testing the threshold hypothesis linking intelligence and creativity. In this study, least–squares fitting was applied to quantify the relationship between ecosystem service values and varying corridor widths. By performing iterative estimations, the optimal breakpoint widths for rural and urban corridors were determined. Empirically determined breakpoints were tested for statistical significance by means of the Davies test^41^. The expression of the piecewise linear function is as follows:

4 $$y={\beta}_{0}+{\beta}_{1}x+\sum_{j=1}^{m-1}{\beta}_{j+1}{(x-{c}_{j})}_{+}+\epsilon$$

where the indicator function ${(x-{c}_{j})}_{+}\mathrm{i}$s defined by:

5 $${(x-{c}_{j})}_{+}=\left\{\begin{array}{c}x-{c}_{j}ifx>{c}_{j}\\0ifx\le{c}_{j}\end{array}\right.$$

Where *y* is the ESV (CNY), representing the monetary valuation of ecological benefits derived from the landscape; *x* is the width of the corridor (m), serving as the explanatory variable that captures the scale of the ecological feature. *β*_*0*_ is the global intercept, indicating the baseline ESV when width is zero and reflecting intrinsic ecological value independent of spatial extent. *β*_*1*_ is the base slope coefficient, describing the initial rate of change in ESV per meter increase in width and representing the marginal contribution of width expansion to ecosystem service value in the smallest landscape. *β*_*j+1*_ is the differential slope parameter for segment (*j + 1*), quantifying changes in the marginal value of width as it surpasses critical thresholds; *c*_*j*_ is the breakpoint where the relationship between width and ESV undergoes a structural shift, marking transitions in corridor scaling ecological service value; the indicator function${(x-{c}_{j})}_{+}$ensures continuity by activating only when width exceeds cⱼ, thereby allowing smooth phase transitions in the modeled relationship; and $\epsilon$ is the random error term, accounting for unexplained variability due to measurement error, model misspecification, or unobserved ecological factors.

## Results

### Land use change

**Fig. 2:**
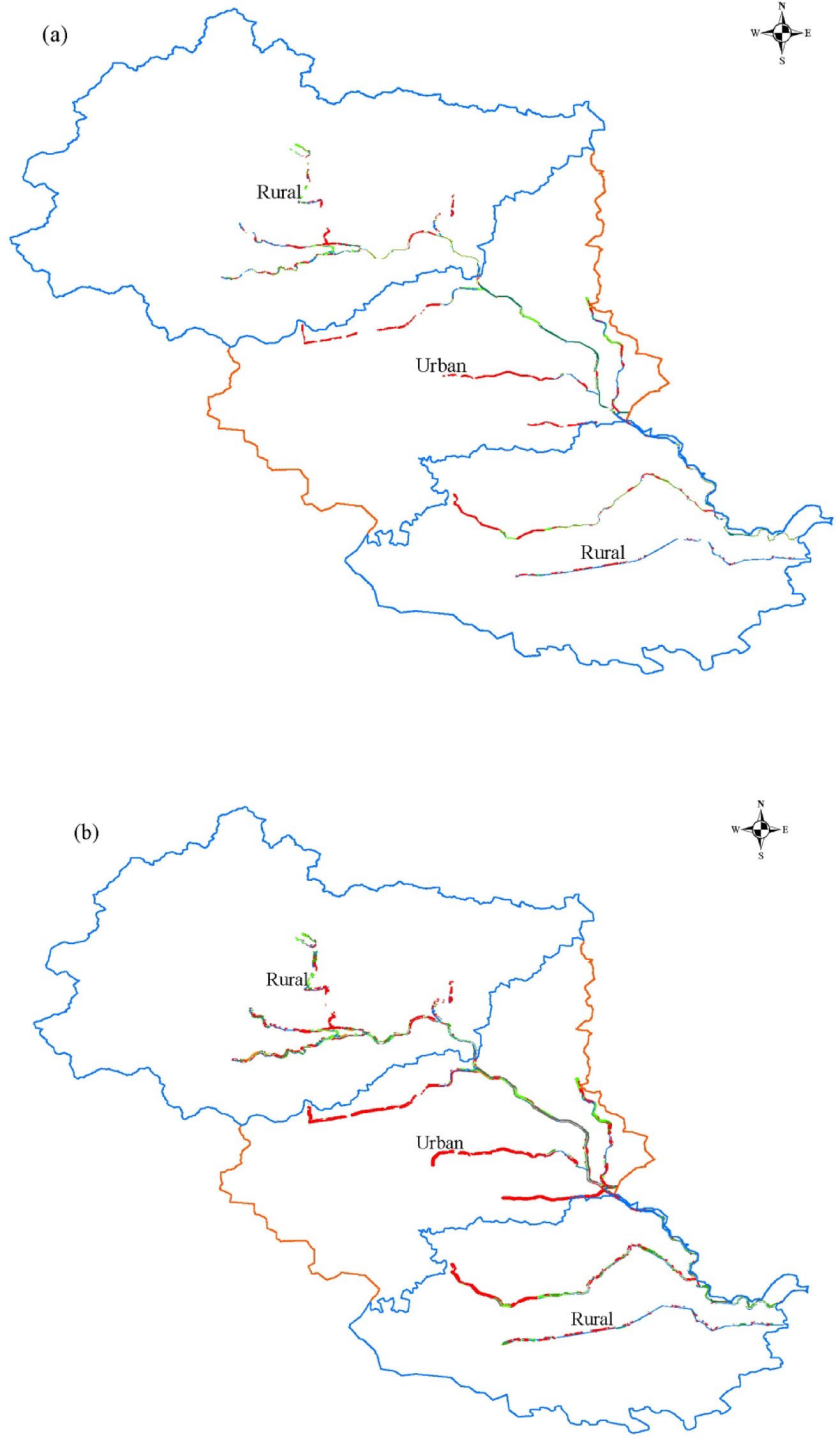

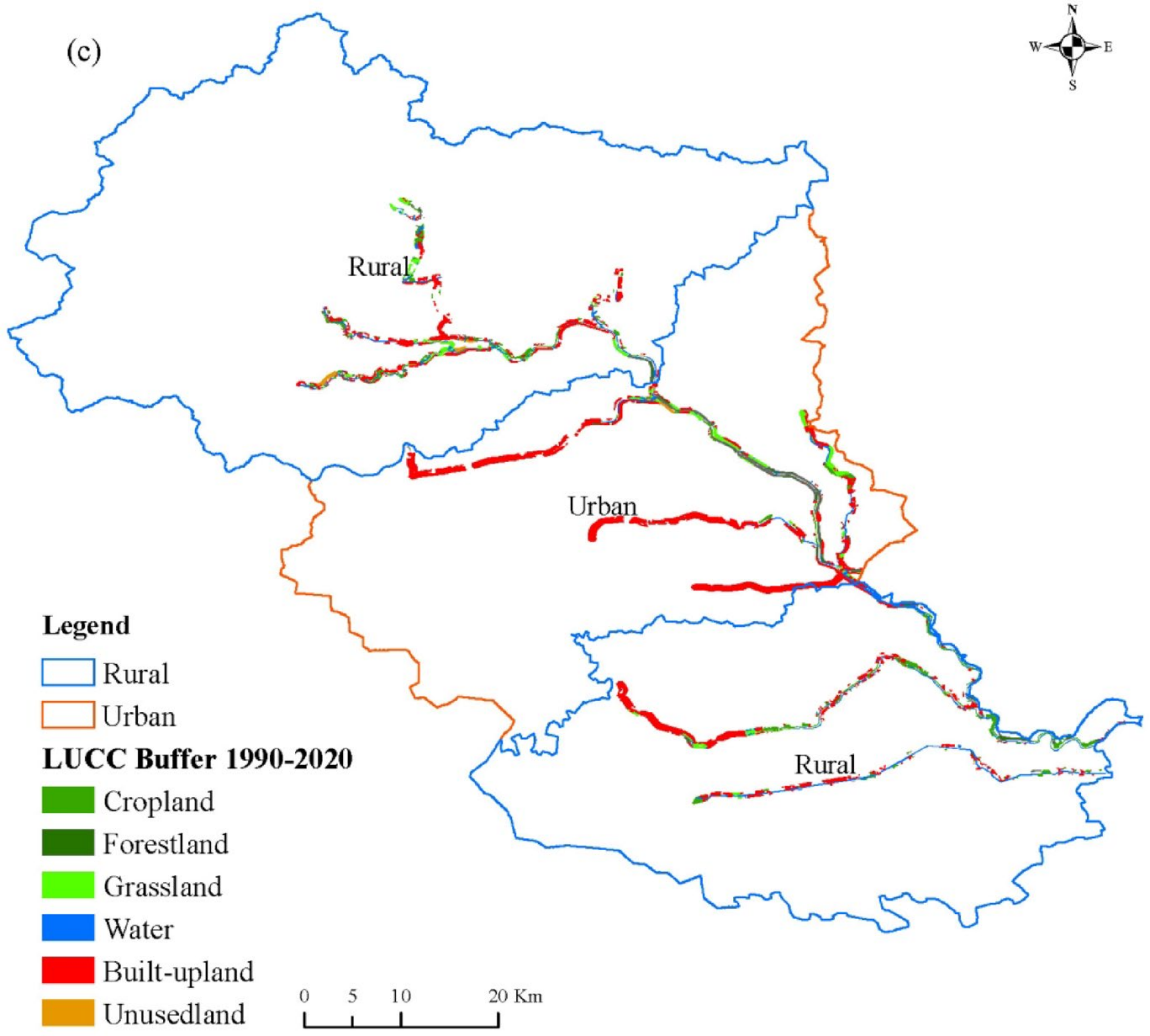
Land–use transfer for rural and urban corridor width of 100 m(**a**), 200 m(**b**), 300 m(**c**) from 1990 to 2020. (Other land–use transfers for rural and urban corridors with additional widths are provided in Supplementary Fig. S1 and Fig. S2.)

As shown in Fig. 2 and Fig.S1–S2, from 1990 to 2020, land–use area within both rural and urban sections of the North Canal River increased with corridor width, while their spatiotemporal trajectories differed significantly (*p* < 0.05). Cropland exhibited a pronounced urban–rural contrast and declined consistently in both corridor types. Over the 30–year period, cropland area decreased by an average of 74.68% in urban corridors and by 35.00% in rural corridors. The major phase of cropland loss occurred, on average, 15 years earlier in urban corridors than in rural corridors. On average, cropland area declined by 36.55% during 1990–1995 in urban corridors, whereas a comparable reduction (36.00%) occurred over the longer period from 1990 to 2010 in rural corridors.

Grassland in the urban sections exhibited a V–shaped change, declining continuously until 2015 before increasing sharply by 2020. For example, within the 300 m corridor, grassland area declined from 45.54 ha in 1990 to 7.68 ha in 2015 and then increased to 450 ha in 2020. In contrast, rural sections exhibited a horizontal Z–shaped pattern, characterized by an initial increase, followed by a sharp decline and subsequent recovery. Grassland area increased slightly by 1995, then declined continuously until 2015, with a maximum reduction of 81.24%, before increasing again thereafter. Within the 300 m corridor, grassland area increased from 441.18 ha in 1990 to 519.06 ha in 1995, declined to 94.80 ha in 2015, and then rebounded to 398.71 ha in 2020.

Forestland exhibited a consistent trajectory in both urban and rural corridors, remaining limited prior to 2020 and then increasing markedly in 2020, with recovery magnitude positively correlated with corridor width. Water bodies displayed distinct spatiotemporal trajectories between urban and rural sections. In urban corridors, water area nearly disappeared by 2010 (only 2.22 ha remaining within the 100 m corridor) but increased substantially by 2020 (reaching 419.94 ha within the 200 m corridor, a 78.5–fold increase relative to 2015). In rural corridors, water area fluctuated only slightly and consistently remained above 650 ha in corridors wider than 500 m.

Built–up land underwent rapid expansion in both corridor types. In urban corridors, expansion accelerated after 2005 and declined after 2015. Built–up land area within the 100 m corridor increased to 689.34 ha by 2005, representing a 205.7% increase. In rural corridors, expansion began after 2010 and peaked in 2015, accounting for 70.20% of total area within the 600 m corridor. By 2020, built–up land still occupied more than 70.00% of total area in rural corridors with widths ≤ 100 m.

The transfer directions of land–use types were consistent across different corridor widths in both rural and urban sections (Table 2). Cropland in rural sections exhibited a relatively high retention rate (mean 54.35%), followed by conversion to built–up land (27.74%). In contrast, cropland in urban sections was primarily converted to built–up land (52.39%), with a much lower retention rate of 21.34%. Grassland displayed divergent transition pathways between rural and urban corridors. In rural sections, grassland was mainly converted to cropland (47.39%), with a retention rate of only 23.89%. In urban sections, grassland was primarily converted to forestland (43.21%), followed by built–up land (23.02%). The dominant transfer directions of forestland, water bodies, and built–up land were broadly consistent between rural and urban sections. Forestland was overwhelmingly converted to built–up land, with transfer rates exceeding 90% in both contexts. Both water bodies and built–up land exhibited relatively high retention rates, being 68.62% and 67.75% in rural sections, and 45.81% and 93.40% in urban sections, respectively. A notable difference was observed in secondary transitions. In urban sections, 23.47% of water bodies were converted to forestland, whereas in rural sections water bodies were mainly converted to built–up land (11.58%) and cropland (10.73%). In addition, 21.09% of built–up land in rural sections was converted back to cropland.

**Table 2:** Mean transfer matrix of land use types at different widths of ecological corridors in rural and urban sections from 1990 to 2020.

Corridors	LUCC	Transfer Matrix(%)					
		Cropland	Grassland	Forestland	Water	Built–up land	Unused land
Rural	Cropland	54.35	4.07	3.52	9.59	27.74	0.73
	Grassland	47.39	23.89	4.07	5.86	18.79	0.00
	Forestland	0.00	0.00	0.00	0.00	100.00	0.00
	Water	10.73	2.72	5.41	68.62	11.58	0.94
	Built–up land	21.09	2.30	3.78	3.85	67.75	1.23
	Unused land	10.03	21.85	7.50	0.42	0.00	60.20
Urban	Cropland	21.34	6.98	8.05	10.97	52.39	0.27
	Grassland	0.00	21.70	43.21	12.07	23.02	0.00
	Forestland	0.00	1.96	0.00	0.00	98.04	0.00
	Water	7.52	23.47	11.21	45.81	11.79	0.20
	Built–up land	0.99	2.42	0.57	2.62	93.40	0.00
	Unused land	9.12	30.18	1.68	0.00	0.00	59.02

### Landscape configuration

Over the three decades from 1990 to 2020, landscape pattern metrics calculated across multiple corridor widths in rural and urban settings showed pronounced spatiotemporal dynamics, reflecting heterogeneous anthropogenic pressures and distinct land–management trajectories along the urban–rural gradient. These configuration metrics provide an integrated representation of structural continuity, fragmentation intensity, and potential landscape connectivity within river corridors. Relative to urban corridors, changes in rural corridor indices were generally more moderate (Table S1), indicating greater long–term structural stability. With increasing corridor width, the largest patch index (LPI), edge density (ED), landscape shape index (LSI), patch fractal dimension (PAFRAC), patch density (PD), and Shannon’s evenness index (SHEI) all declined, whereas the contagion index (CONTAG) increased, particularly in rural corridors. This systematic shift reflects a transition from fragmented, edge–dominated configurations toward more aggregated and structurally coherent landscapes. For example, the maximum coefficients of variation for LPI and ED at the 100– and 600–m widths reached 27.11% and 4.35%, respectively. In 2020, PD for the 600–m corridor (2.60) was 78.6% lower than that for the 100–m corridor (3.21), and CONTAG for the 600–m corridor (60.51) was 4.28% higher than for the 100–m corridor (56.23). These patterns indicate that increasing corridor width enhances landscape aggregation and reduces edge effects, thereby strengthening overall structural stability and the potential for maintaining lateral ecological connectivity.

**Fig. 3:**
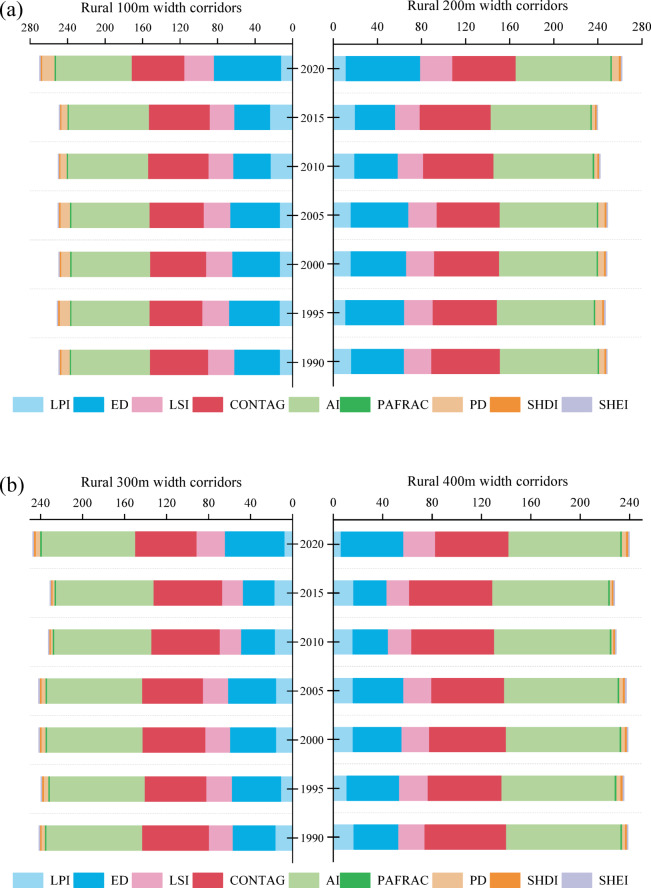

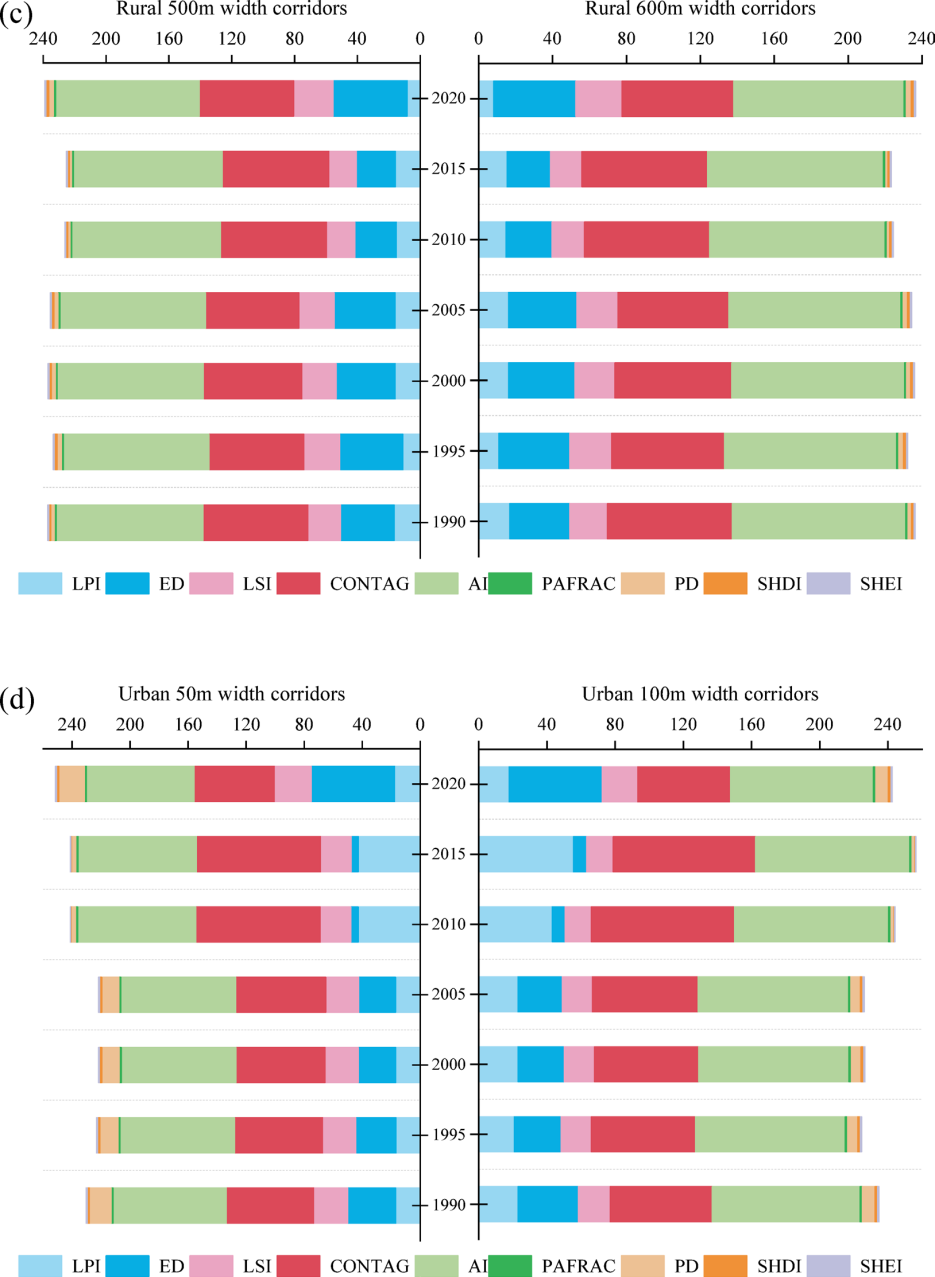

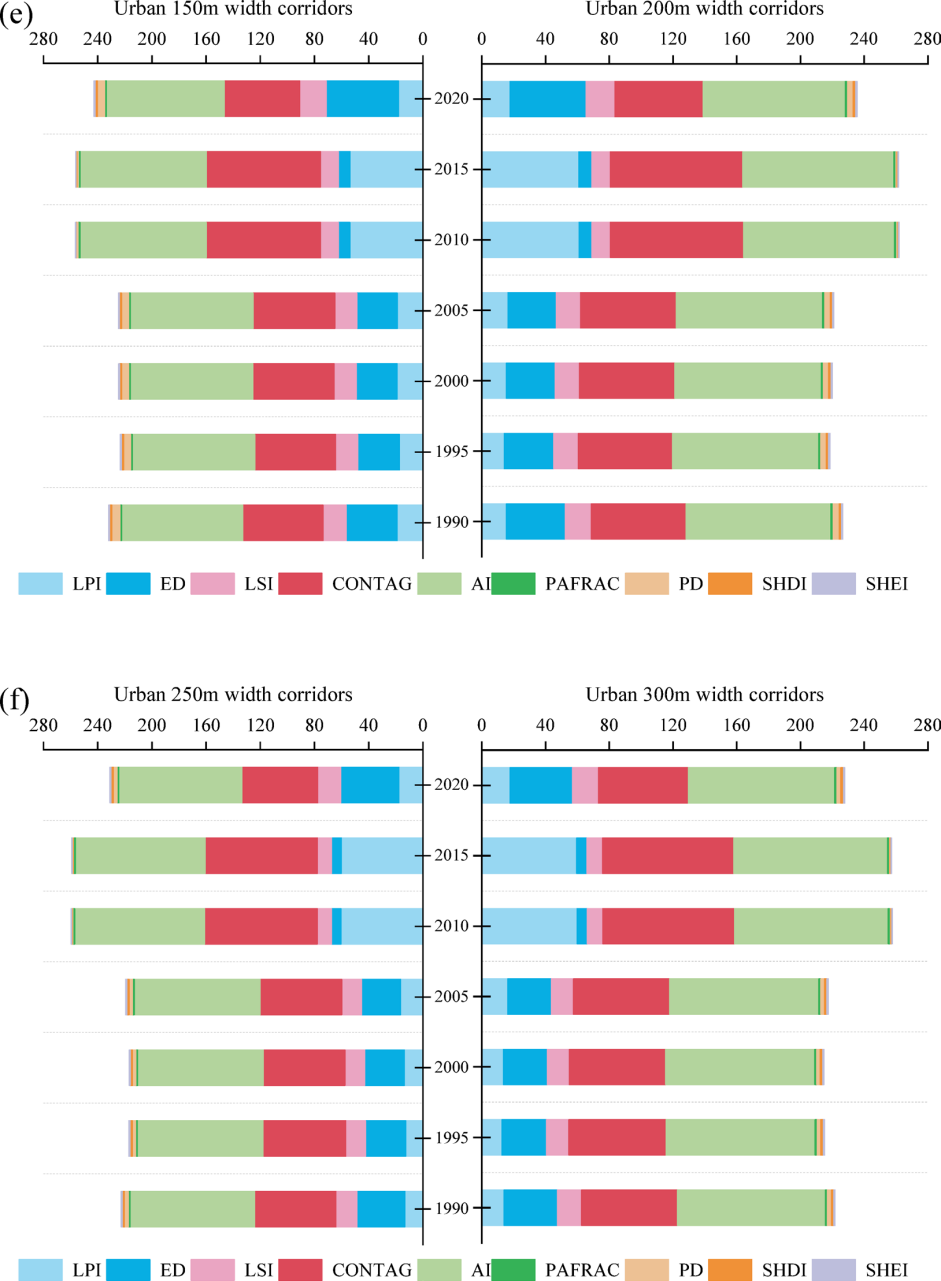
Landscape pattern index for different corridors width of rural and urban from 1990 to 2020. (From (**a**)–(**c**), Landscape pattern index for rural corridor width at 100 m, 200 m, 300 m, 400 m, 500 m, and 600 m, respectively. (**d**) – (**f**) Landscape pattern index for urban corridor width at 50 m, 100 m, 150 m, 200 m, 250 m, and 300 m, respectively.)

As illustrated in Fig. 3, interannual trajectories of landscape metrics were broadly consistent across widths within both corridor types. In rural corridors (Fig. 3a and c), CONTAG attained a peak of 67.75 at the 600–m width during 2010–2015, while the aggregation index (AI) remained consistently high (> 90%), suggesting a relatively intact and connected landscape structure. Landscape heterogeneity increased after 2015, coinciding with accelerated land–use transitions. By 2020, PD at 200 m width had doubled relative to 2015, increasing from 2.80 to 6.34. Shannon’s diversity index (SHDI) remained greater than 1.70 for corridors wider than 300 m. Integrating these configuration changes with spatiotemporal land–use dynamics indicates that the post–2010 expansion of built–up land was a primary driver of renewed fragmentation and increasing heterogeneity in rural corridors.

Urban corridors exhibited a contrasting temporal signature characterized by pronounced homogenization around 2010 (Fig. 3d and f). Between 1990 and 2010, ED declined sharply at all widths: reductions were 84.72%, 78.72%, 77.42%, 78.30%, 79.81%, and 80.53% for widths of 50, 100, 150, 200, 250, and 300 m, respectively (mean decrease 79.97%). Over the same period, LPI increased to 2.32 times its initial value, while SHDI decreased to 0.50, indicating the dominance of built–up land and a highly simplified landscape structure. From 2010 to 2020, ED increased markedly across all widths (mean increase 616.53%), coincident with expansion of blue–green spaces such as grassland, forestland, and water bodies under large–scale restoration initiatives. During this recovery phase, mean LPI declined from 53.55% to 17.85%, and mean SHDI rose from 0.43 to 1.59, reflecting partial structural diversification of urban corridors. Despite these improvements, narrow urban corridors (≤ 100 m) remained highly fragmented in 2020, with CONTAG below 56.47 and AI below 83.76, indicating persistent edge dominance and limited structural connectivity. Overall, these results demonstrate a strong width effect on landscape configuration and structural stability in both rural and urban contexts. Wider corridors were consistently associated with more aggregated, less edge–dominated landscapes, while the timing and magnitude of homogenization and subsequent recovery differ between settings, consistent with distinct land–use pressures and ecological restoration trajectories along the urban–rural continuum.

### Changes in ESV

To evaluate the robustness of ESV estimates to uncertainty in valuation coefficients (VCs), the coefficient of sensitivity (CS) was calculated for rural and urban subbasins for the period 1990–2020 (Table S2). Across all land–use types and years, CS values remained below one, indicating low sensitivity of ESV estimates to variation in VCs. In rural subbasins, cropland (0.13–0.21) and water (0.16–0.30) exhibited moderate and stable sensitivity, whereas grassland consistently showed the highest sensitivity (0.53–0.64). In urban subbasins, sensitivity of built–up land increased from 0.42 in 1990 to 0.84 in 2015 and then declining to 0.72 by 2020. In contrast, the sensitivity of urban water bodies declined from approximately 0.28 during 1990–2000 to less than 0.10 after 2010, reflecting conversion of aquatic areas to built–up land. Despite these shifts, all CS values remained below one, confirming that the valuation–coefficient framework of Xie et al.,^29^ yields stable and credible ESV estimates. These findings align with prior work reporting similar robustness in structurally stable landscapes^33, 34^.

**Fig. 4:**
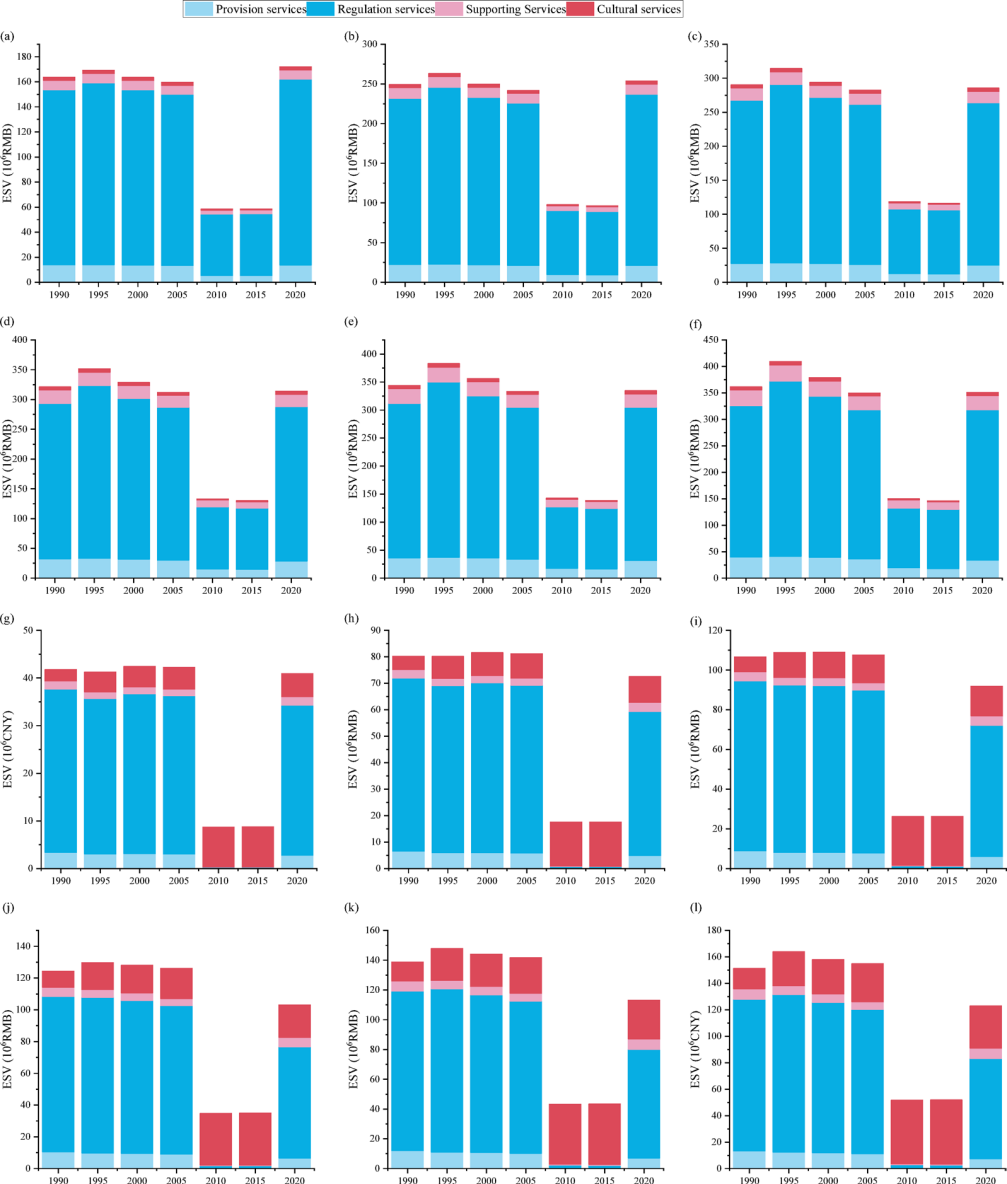
ESV for corridor width of rural and urban from 1990 to 2020. (From (**a**)–(**f**), ESV for rural corridor width at 100 m, 200 m, 300 m, 400 m, 500 m, and 600 m, respectively. (**g**) – (**l**) ESV for urban corridor width at 50 m, 100 m, 150 m, 200 m, 250 m, and 300 m, respectively.)

The total ESV along the Beijing section of the North Canal River corridor exhibited a pronounced urban–rural divergence from 1990 to 2020 (Fig. 4). In rural corridors (Fig. 4a and f), provision services dominated in the early period, cropland–derived food provision reaching 1.58 million CNY in 1990 (approximately 60% of total provision services value) but declining by 41% to 0.93 million CNY by 2010 due to farmland conversion. Water–related provision decreased by about 65% over the same period, from 0.97 million CNY in1990 to 0.34 million CNY in 2010, together accounting for a 51% reduction in rural provision ESV. Supporting services, primarily habitat and soil fertility comparatively stable until 2005 but contracted by approximately 65% thereafter (Hab_Water declined from 3.10 million CNY in 1990 to 1.08 million CNY in 2010). Consequently, the rural landscape shifted from a provision– and habitat–dominated structure toward a more regulation–focused but overall smaller ESV level.

In contrast, urban corridors displayed an opposite pattern (Fig. 4g and l). Provision services declined sharply as cropland and water bodies decreased. Urban FP_total dropped from 0.63 million CNY in 1990 to < 0.06 million CNY in 2010, representing a loss exceeding 90%). Meanwhile, cultural services increased markedly. In the 50 m urban corridor, cultural ESV rose from 2.42 million CNY in 1990 (about 65% of total urban ESV) to 8.35 million CNY in 2010, representing an increase of approximately 245%. Habitat services in urban corridors remained consistently low (< 0.85 million CNY) throughout the study period.

Based on the specified width gradients (urban: 50–300 m; rural: 100–600 m), ESV exhibited a nonlinear relationship with corridor width. In urban corridors (Fig. 4g and l), total ESV increased from 41.79 million CNY at 50 m to 151.37 million CNY at 300 m in 1990, representing a total increase of 262.20%. Incremental gains declined stepwise with each 50 m expansion, being 91.95% from 50 to 100 m, 33.05% from 100 to 150 m, 16.57% from 150 to 200 m, 11.59% from 200 to 250 m, and 9.04% from 250 to 300 m. A similar pattern was observed in later years. In 2010, the increment from 50 to 100 m (101.7%) substantially exceeded that from 250 to 300 m (19.40%), and in 2020 the corresponding increments were 77.29% and 8.56%, respectively. Collectively, these results suggest that in urban corridors most ESV gains are realized within widths up to approximately 150–200 m, beyond which the additional benefit per 50 m expansion falls below roughly 20%.

Rural corridors exhibited a comparable but broader response range (Fig. 4a and f). In 1990, total ESV increased from 163.82 million CNY at 100 m to 361.87 million CNY at 600 m, with progressively smaller increments of 52.12% from 100 to 200 m, 16.49% from 200 to 300 m, 10.69% from 300 to 400 m, 7.10% from 400 to 500 m, and 5.15% from 500 to 600 m. Similar attenuation was observed in 2010 (67.53% from 100 to 200 m compared with 20.74% from 200 to 300 m) and in 2020 (47.46% from 100 to 200 m compared with 12.55% from 200 to 300 m). Together, these trajectories indicate a consistent width–dependent pattern across both corridor types, with diminishing marginal ESV gains occurring at narrower widths in urban corridors (approximately 150–200 m) than in rural corridors (approximately 200–300 m).

### The optimal width of rural and urban corridors

**Fig. 5:**
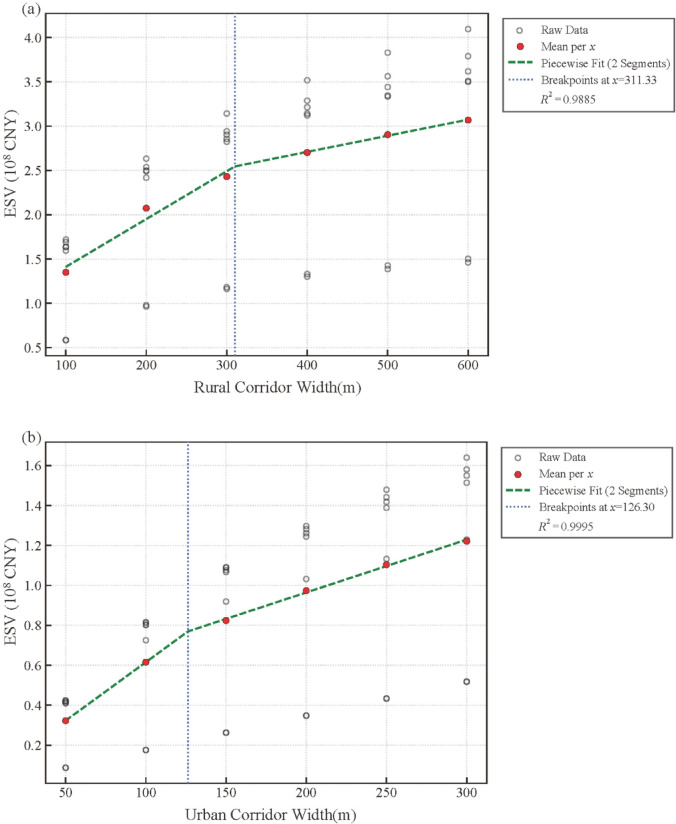
Breakpoint models for the widths of rural corridors (**a**) and urban corridors (**b**).

To quantify threshold–like responses and identify specific width breakpoints, segmented regression analysis was applied to the relationships between corridor width and ESV for rural and urban sections. For rural corridors, a significant breakpoint was identified at a width of 311.33 m, as confirmed by the Davies test for slope change (*p* < 0.05). Below this threshold, ESV demonstrated a strong positive response to width increase, with each additional meter of width corresponding to an average gain of approximately 8.79 million CNY in ESV. Beyond 311.33 m, the slope of the relationship declined markedly, indicating a pronounced reduction in the rate of ESV increase. This transition suggests that once a certain spatial extent is reached, additional corridor widening contributes progressively less to overall ecosystem service output. The segmented model exhibited a very high explanatory power (R² > 0.98), accounting for more than 98% of the observed variance in rural ESV (Fig. 5a). Urban corridors exhibited a comparable but more compressed non–linear response. A significant breakpoint was identified at 126.30 m (*p* < 0.05), separating a steep initial phase from a flatter response segment. Below this width, ESV increased sharply with corridor expansion, reflecting the strong sensitivity of urban ecosystem services to incremental increases in corridor space. Beyond 126.30 m, the rate of increase declined substantially, indicating that the marginal contribution of additional width weakened considerably. The urban segmented regression model achieved an excellent fit (R^2^ > 0.99), confirming the robustness of the detected breakpoint and its strong explanatory power for ESV variation (Fig. 5b).

Comparatively, the rural breakpoint occurred at a substantially greater width than the urban breakpoint, indicating that wider spatial extents are required in rural landscapes before ESV responses approach saturation. In contrast, urban corridors reached the zone of diminishing returns at much narrower widths, highlighting fundamental differences in the scaling behavior of ecosystem services between highly urbanized and less disturbed river environments. Together, these results demonstrate that the ESV–width relationship in both corridor types follows a threshold–like pattern characterized by rapid initial gains followed by attenuated responses, while the position of the threshold varies systematically along the urban–rural gradient.

## Discussion

### Divergent land use transitions and landscape dynamics along the urban–rural gradient

The North Canal River corridor exhibits strong spatial heterogeneity shaped by urban expansion and differential land–use trajectories^42, 43^. However, river corridors are not merely static landscape units, but dynamic systems structured by interacting hydrological, geomorphological, and ecological processes. Corridor width therefore represents not only a planning boundary, but also a proxy for the functional space required to sustain key riverine processes, including lateral hydrological connectivity, sediment exchange, and habitat maintenance^25^; ^44^. Along the North Canal River, rural segments remain dominated by cropland, water bodies and semi-natural patches that supptort regulation and supporting services, such as climate regulation, soil retention, and habitat provision^9^. These services are underpinned by relatively intact floodplain connectivity, higher infiltration capacity, and greater continuity of riparian vegetation, which facilitate material, water, and nutrient exchange between river channel and its surrounding landscape (O’Briain, 2019;^54,^ ^6^. In contrast, urban segments are characterized by extensive built-up land, channelization, and patch fragmentation, which constrain hydro–morphological processes, disrupt lateral river–floodplain interactions, and weaken ecological flows that sustain regulating and supporting services^45, 44, 4^. These patterns are consistent with the emerging pattern–process–services–sustainability paradigm, which emphasizes the causal linkages between spatial heterogeneity, ecological processes, service provision, and long–term sustainability^11, 10^. Within this paradigm, corridor width emerges a critical spatial attribute because it directly constrains the space available for overbank flooding, sediment deposition, nutrient retention, and riparian succession, thereby influencing hydro-morphological quality and ecological integrity^45, 44^.

Landscape metrics further reinforced this differentiation: rural segments retained higher patch diversity and ecological connectivity, conferring resilience against land conversion pressures, while urban segments showed pronounced homogenization around 2010, with only partial recovery after large–scale restoration efforts such as the “Blue–Green Space Plan” (2017) and riparian ecological rehabilitation projects covering the main urban area initiated by the Beijing Municipal Government since 2013. This suggests that maintaining landscape heterogeneity is a critical precondition for the resilience of social–ecological systems in megacity regions^46^.

### Trade–offs and resilience of ecosystem services across river corridor widths

The responses of ESV to corridor width reveal fundamentally different functional behaviors between rural and urban corridors, reflecting pronounced spatial contrasts in hydro-morphological conditions and service provision. In rural corridors, relatively stable cropland, forest, and grassland mosaics support persistent regulation and supporting services, including climate regulation, water regulation, and erosion control. These services are closely associated with intact floodplain connectivity, riparian continuity, and infiltration capacity (Fig. 3), which enable sustained material and energy exchange between channels and surrounding landscapes (O’Briain, 2019:^54,^^44, 6^. By contrast, urban corridors exhibit pronounced service reconfiguration. With extensive cropland loss, provisioning services declined sharply, whereas cultural services expanded rapidly as riverfront spaces were redeveloped for recreation and aesthetic functions (Fig. 3). However, regulating and habitat services showed only limited recovery, consistent with the continued simplification and fragmentation of urban landscapes. Studies by Kang et al., (2025a,^47^ indicated that local-scale fragmentation and habitat simplification could offset potential service gains, reinforcing that corridor widening alone cannot compensate for degraded spatial structure and vegetation condition.

This trade–off is consistent with empirical work showing that expansion of built–up land is a primary driver of ESV loss at regional scales^48^ and aligns closely with Beijing’s planning priorities. The Master Plan of Development for Beijing (2016–2035) emphasizes the integration of green and blue infrastructure to enhance livability in dense urban zones^49^. Meanwhile, the Ecological Conservation Red Line policy protects rural river corridor as critical spaces for water conservation, biodiversity, and ecological security^50^. The resilience of rural ESV highlights their buffering role in maintaining multifunctionality, while the partial recovery of urban regulating services by 2020 through initiatives including wetland park construction and riparian vegetation rehabilitation demonstrates the potential of restoration to counterbalance urbanization pressures^33, 34^.

Beyond composition, landscape configuration and hydro–morphological space jointly modulate how river corridor width translates into ecosystem services. As corridor width increases, the incorporation of larger floodplain areas initially reduces landscape fragmentation, weakens edge effects, and improves patch aggregation, as reflected by declining patch density and rising CONTAG values (Fig. 3). These configuration shifts enhance lateral hydrological connectivity, riparian continuity, and habitat availability, resulting in rapid gains in regulating and supporting services. However, once a basic functional river space is established, further corridor widening produces progressively smaller changes in landscape configuration and connectivity. Key processes such as channel–floodplain exchange, sediment retention, and habitat capacity approach saturation, and additional width contributes proportionally less to service provision^44, 6, 25^. Our analyses indicate that narrow river corridors (< 100 m) often remain highly fragmented and edge–dominated, particularly in urban contexts, limiting the ability to sustain regulating and supporting services. Intermediate to wide corridors (> 200–300 m) substantially improve structural connectivity and ecological function, but beyond this range, marginal gains diminish as landscape configuration and process capacity stabilize. This width–dependent scaling behavior is consistent with broader findings on non–linear scale effects in hydrological regulation, water purification, and soil conservation across spatial extents^14^.

### Implications for ecological corridor planning and optimal width determination

The segmented regression analysis identified distinct service–based width thresholds for rural corridors at 311.33 m and for urban corridors at 126.30 m, beyond which ESV gains diminished. These thresholds are not merely statistical breakpoints but fall within the range of functional river space and active floodplain extents reported in previous fluvial studies. Empirical evidence indicates that key hydro-geomorphological processes, including overbank flooding, sediment exchange, and riparian succession, are typically concentrated within several tens to several hundreds of meters from the channel, corresponding to the active floodplain and historical migration zones of lowland rivers^45, 25^; O’Briain, 2019;^4^. The close correspondence between these functional spatial ranges and the thresholds in this study suggests that the non-linear ESV responses reflect the progressive saturation of riverine process space rather than purely geometric scaling effects. Moreover, the pronounced difference between rural and urban thresholds further highlights the functional heterogeneity of ecosystem services along the urban–rural gradient, which is consistent with emerging concepts of ecosystem service scaling that emphasize the sensitivity of service provision to spatial configuration and scale^11, 51^. Together, these findings underscore the need for spatially adaptive corridor design that accounts for differential ecosystem responses across landscape contexts.

From a planning perspective, the results provide quantitative, service-oriented references for river corridor design rather than uniform width prescriptions. Integrating width thresholds derived from ecosystem service responses into river corridor planning frameworks can support the translation of ecological targets into spatially explicit management guidance. In urban settings, where land availability is limited, planning should prioritize restoring channel–floodplain connections, enhancing riparian structural complexity, and improving vegetation condition within constrained spaces, rather than focusing solely on corridor widening. In rural landscapes, safeguarding sufficiently wide corridors remains essential for preserving hydro-morphological dynamics, buffering flood pulses, and maintaining large-scale ecological connectivity that sustains long-term multifunctionality and resilience^52^; O’Briain, 2019;^54,^ ^4^. This approach aligns with recent recommendations advocating the integration of spatial configuration metrics into river corridor design algorithms to enhance ecological functionality and service provision^22^. By explicitly linking corridor width to ecosystem service outcomes and underlying ecological processes, this study contributes to operationalizing the final link in the pattern–process–services–sustainability paradigm. The proposed framework offers a transferable approach for embedding ecosystem service responses into spatial river corridor planning, supporting adaptive management under ongoing urbanization and climate change pressures.

Nevertheless, the valuation of ecosystem services in this study is regionally specific. The North Canal River Basin, embedded within a megacity context, exhibits distinctive land-use structures and management objectives that influence both the selection of value coefficients and the monetization of regulatory and cultural services. Consequently, the numerical thresholds reported here should be interpreted as planning-oriented reference values rather than universal standards. Sensitivity analysis confirms the robustness of overall response patterns, supporting the internal consistency of the framework. Future research should integrate additional biophysical and ecological indicators, biodiversity data, and long-term monitoring records, together with explicit simulation of key hydrodynamic processes such as flood frequency, sediment transport, and surface water–groundwater interactions. Such integration will further strengthen mechanistic understanding and enhance the transferability of optimized corridor width recommendations across diverse fluvial systems.

## Conclusions

Landscape patterns and ecosystem services in river corridors have been widely studied, yet width-dependent effects along the urban–rural gradient and the implications of valuation uncertainty remain underexplored. Using the North Canal River corridor as a case, we combined multi–temporal land–use data (1990–2020), landscape metrics, and ecosystem service value (ESV) assessments to build a service–oriented framework for corridor width optimization. The conclusions and proposals were drawn as follows:

Clear functional divergence was evident along the urban–rural gradient of the urban river. In urban segments, rapid expansion of built–up land produced marked homogenization around 2010, accompanied by steep declines in provision services and a shift toward cultural services; subsequent blue–green restoration yielded only partial recovery. Rural segments retained continuous cropland, grassland, and water, sustaining regulation and supporting services and preserving higher connectivity. Quantitatively, cropland within urban corridors decreased by roughly three quarters over three decades, compared with about one third in rural corridors. Urban edge density declined by about 80% by 2010 and later rebounded with restoration, while landscape diversity in urban areas rose to approximately 1.6 by 2020. ESV responded nonlinearly to corridor width, with diminishing marginal gains beyond small–to–intermediate bands. Most urban benefits accrued within roughly 100–150 m, whereas rural corridors continued to generate substantial gains up to about 200–300 m. Segmented regression identified thresholds near 126 m for urban corridors and 311 m for rural corridors, beyond which added width conferred limited incremental value. These findings provide a defensible basis for differentiated service–based width standards consistent with the Master Plan of Development for Beijing (2016–2035) and the Beijing Garden City Special Planning (2023–2035). By integrating the pattern–process–service–sustainability framework with actionable spatial thresholds, the study offers an operational pathway for precision river corridor width design that can enhance ecological resilience and support adaptive management of megacity river systems.

## Supplementary Information

Below is the link to the electronic supplementary material.

Supplementary Material 1

## Data Availability

The datasets used and analyzed during the current study are available from the corresponding author on reasonable request.
