# Supplementary material for: Segmented optimization for river ecological corridor width based on ecosystem services: a case study of the North Canal River, China
Source: Sci Rep. 2026 Mar 22;16:14524. doi: 10.1038/s41598-026-43293-2 (PMC13150018; doi:10.1038/s41598-026-43293-2)
Supplement: Supplementary file 1 — Supplementary Material 1 [file 41598_2026_43293_MOESM1_ESM.docx]

**Supplementary Materials**

**Segmented optimization for river ecological corridor width based on ecosystem services: a case study of the North Canal River, China**

Liying Zhu^1,2^, Yuansong Wei^1,2,3*^, Jie Zhao^4^, Dawei Yu^1,2^, Wenjing Zhang^5^, Min Yan^6^, Xinyuan Xu^1,2^

^1.^ State Key Laboratory of Regional Environment and Sustainability, Research Center for Eco-Environmental Sciences, Chinese Academy of Sciences, Beijing 100085, China

^2.^ Laboratory of Water Pollution Control Technology, Research Center for Eco-Environmental Sciences, Chinese Academy of Sciences, Beijing 100085, China

^3.^ University of Chinese Academy of Sciences, Beijing 100049, China

^4.^ Beijing Municipal Institute of City Planning & Design, Beijing 100045, China

^5.^ Chinese Academy of Environmental Planning, Ministry of Ecology and Environment, Beijing, 100041, China

^6.^ Key Laboratory of Digital Earth Science, Aerospace Information Research Institute, Chinese Academy of Sciences, Beijing 100094, China

^*^ Corresponding Author, Yuansong Wei, ([yswei@rcees.ac.cn](mailto:yswei@rcees.ac.cn))

**Contents**

**Figures**

Fig.S1 Land-use transfer for urban corridors with width of 50m(a), 150m(b), 250m(c) from 1990 to 2020.

Fig.S2 Land-use transfer for rural corridors with width of 400m(a), 500m(b), 600m(c) from 1990 to 2020.

**Tables**

Table S1 Landscape Pattern Indices of Rural and Urban Ecological Corridors from 1990 to 2020

Table S2 Sensitivity index of ecosystem service-function values and land uses in the North Canal River basin from 1990 to 2020.


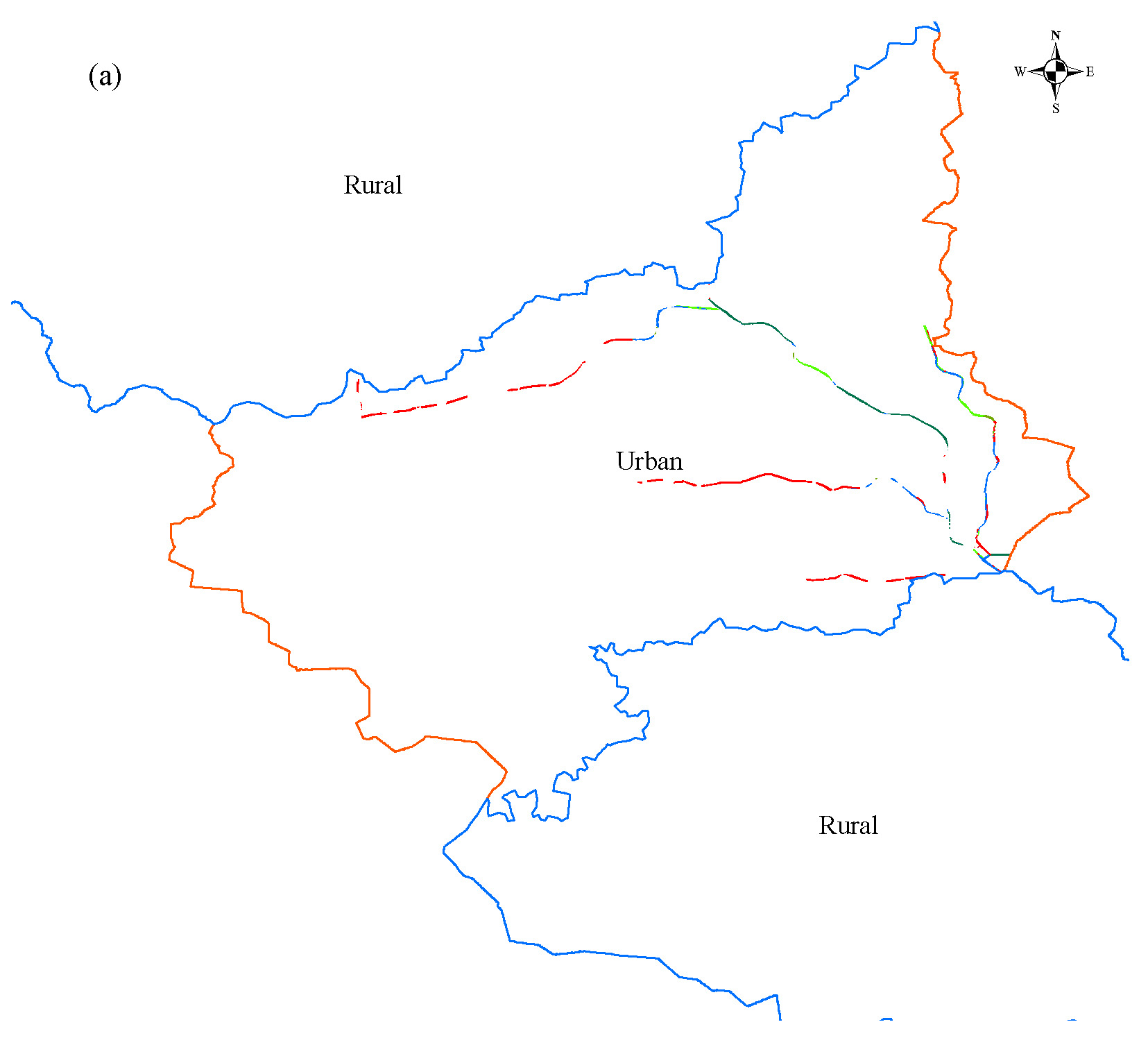

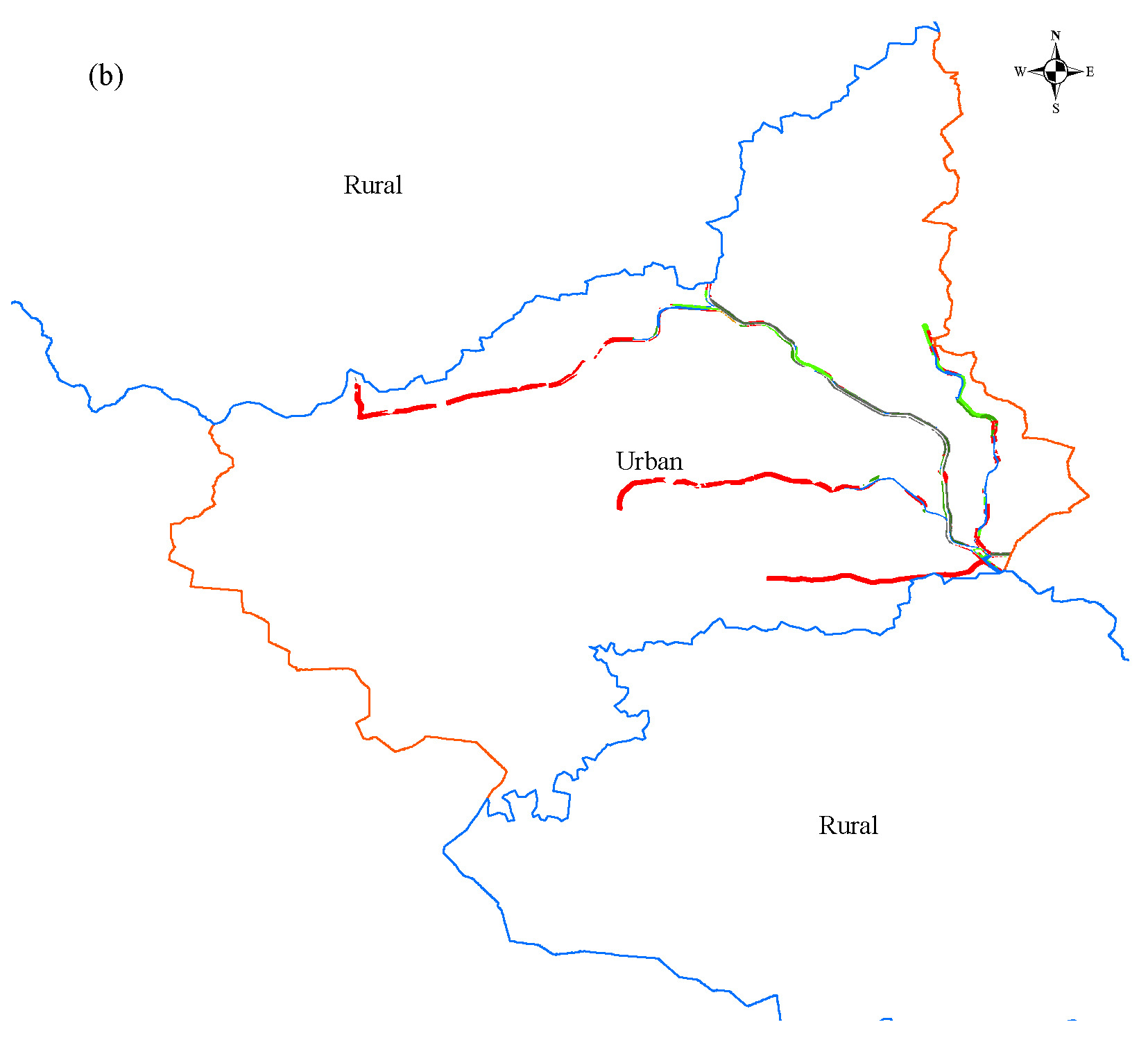

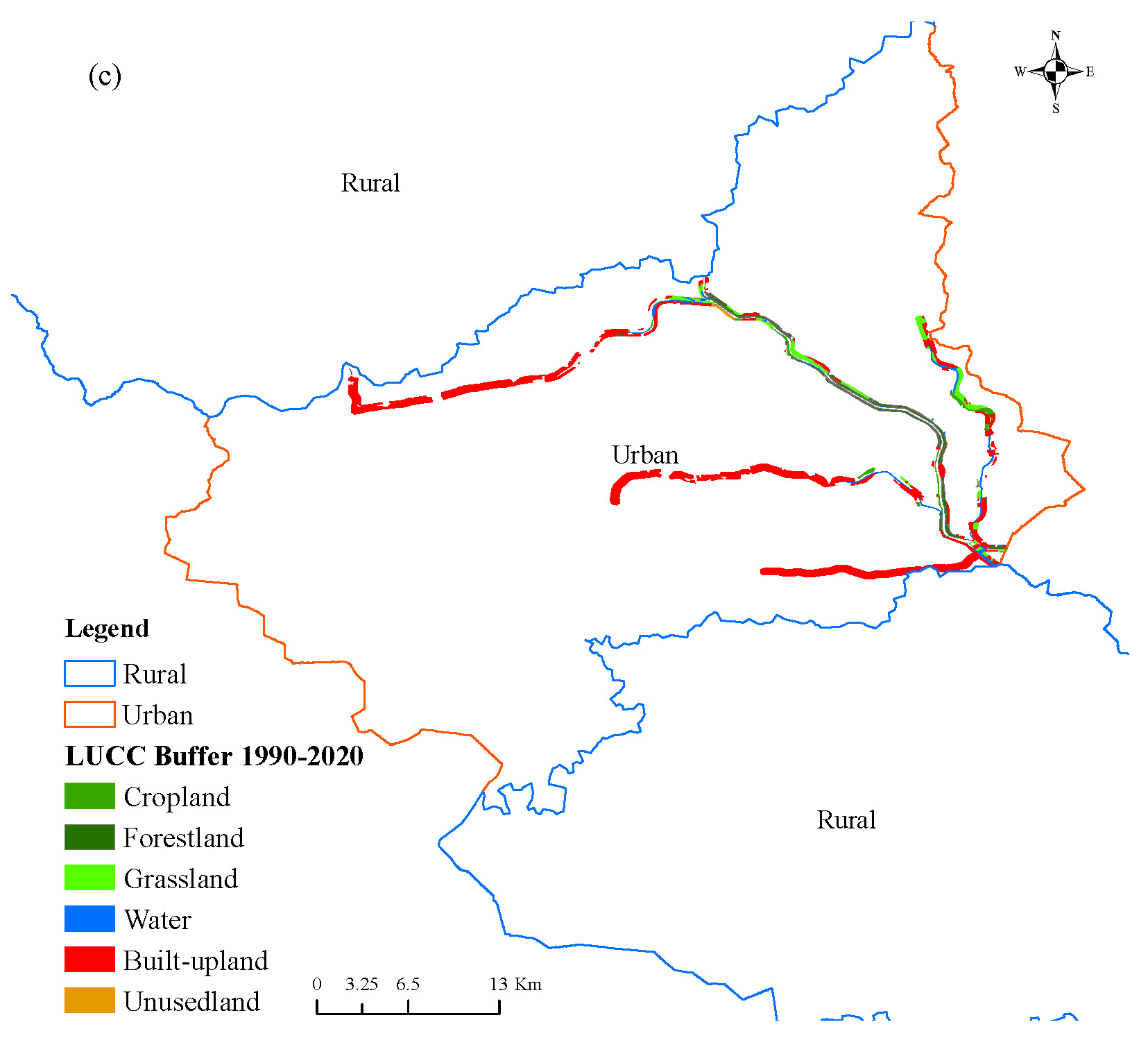


Fig. S1 Land-use transfer for urban corridors with width of 50m(a), 150m(b), 250m(c) from 1990 to 2020.


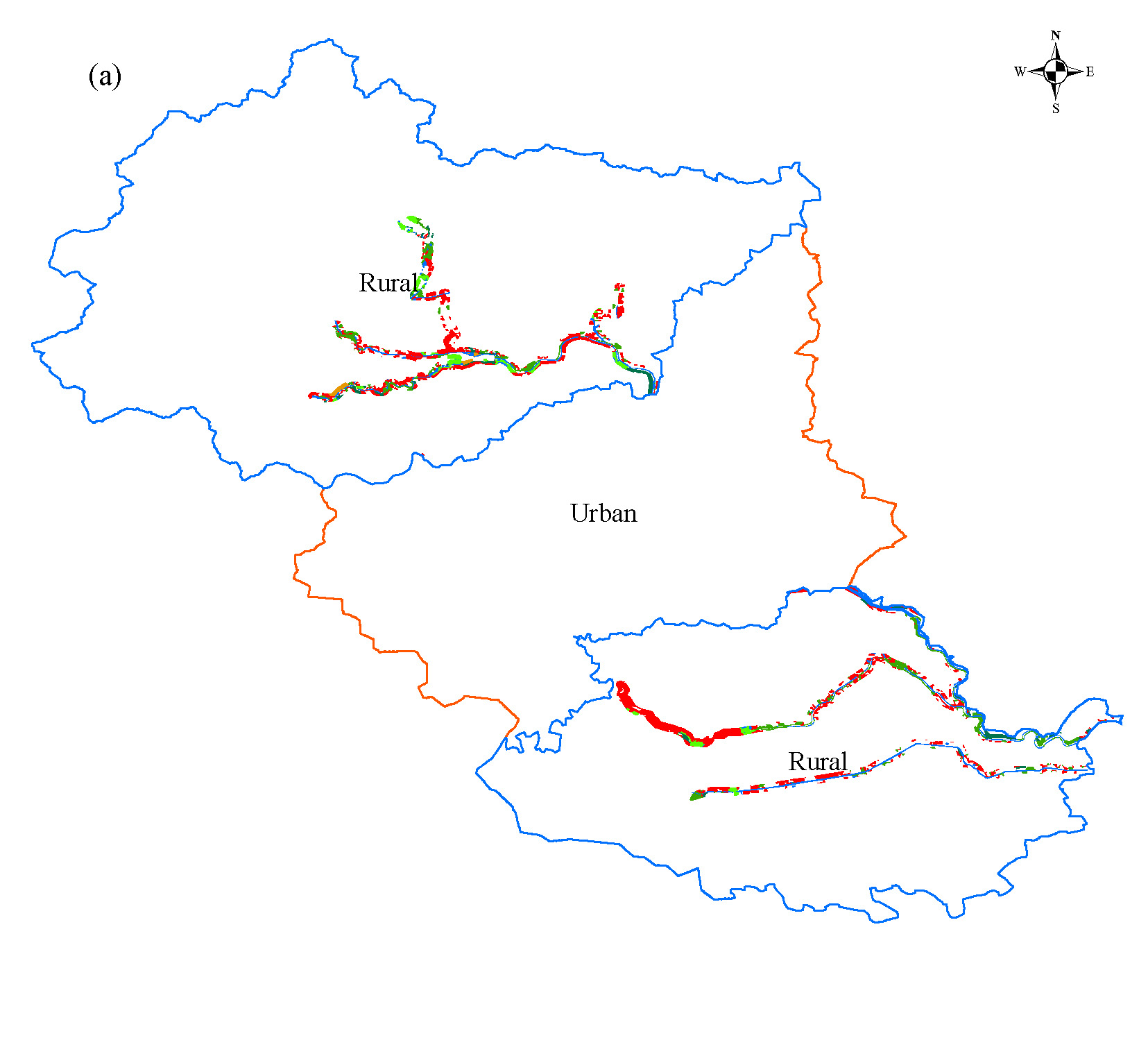

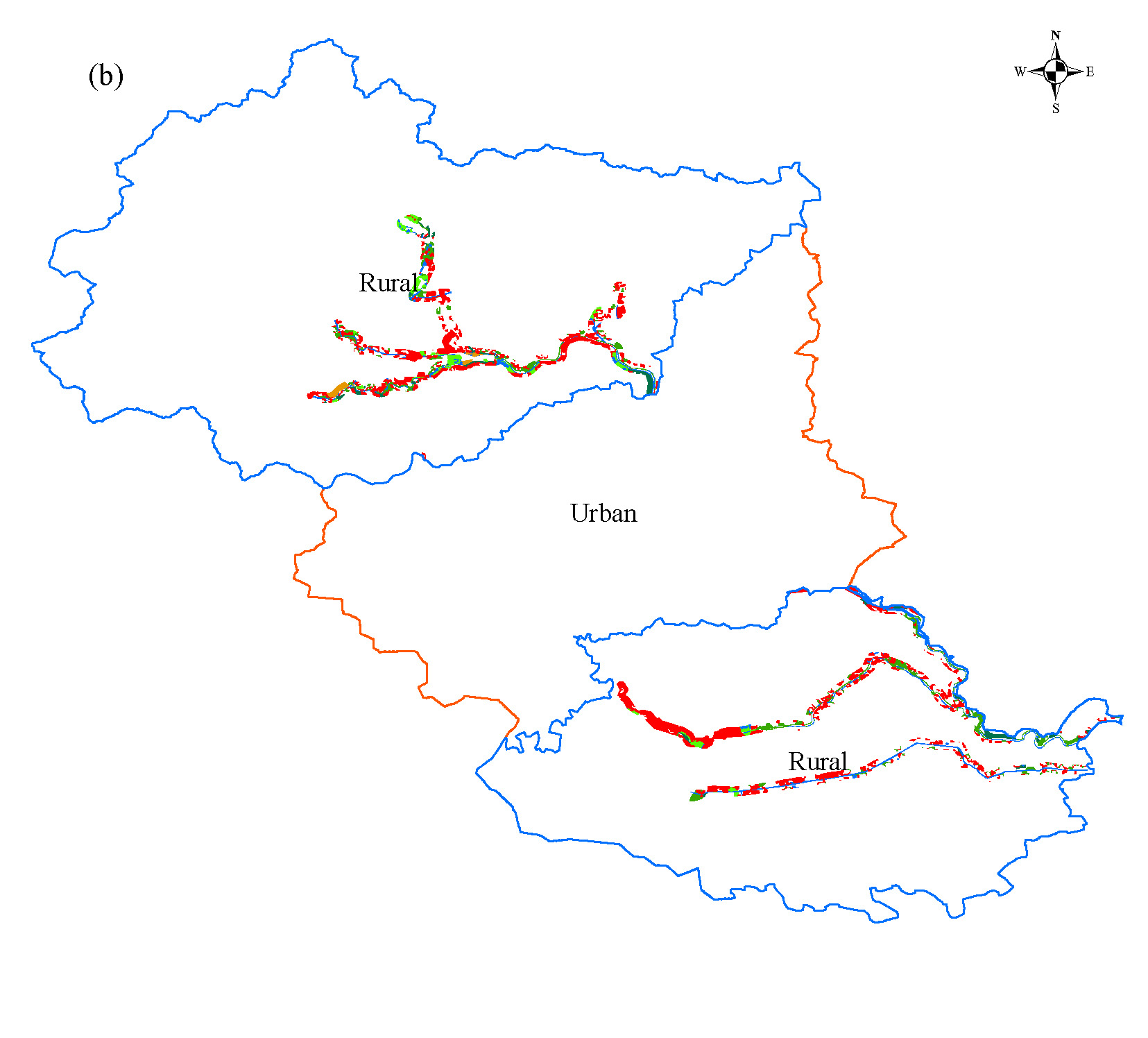


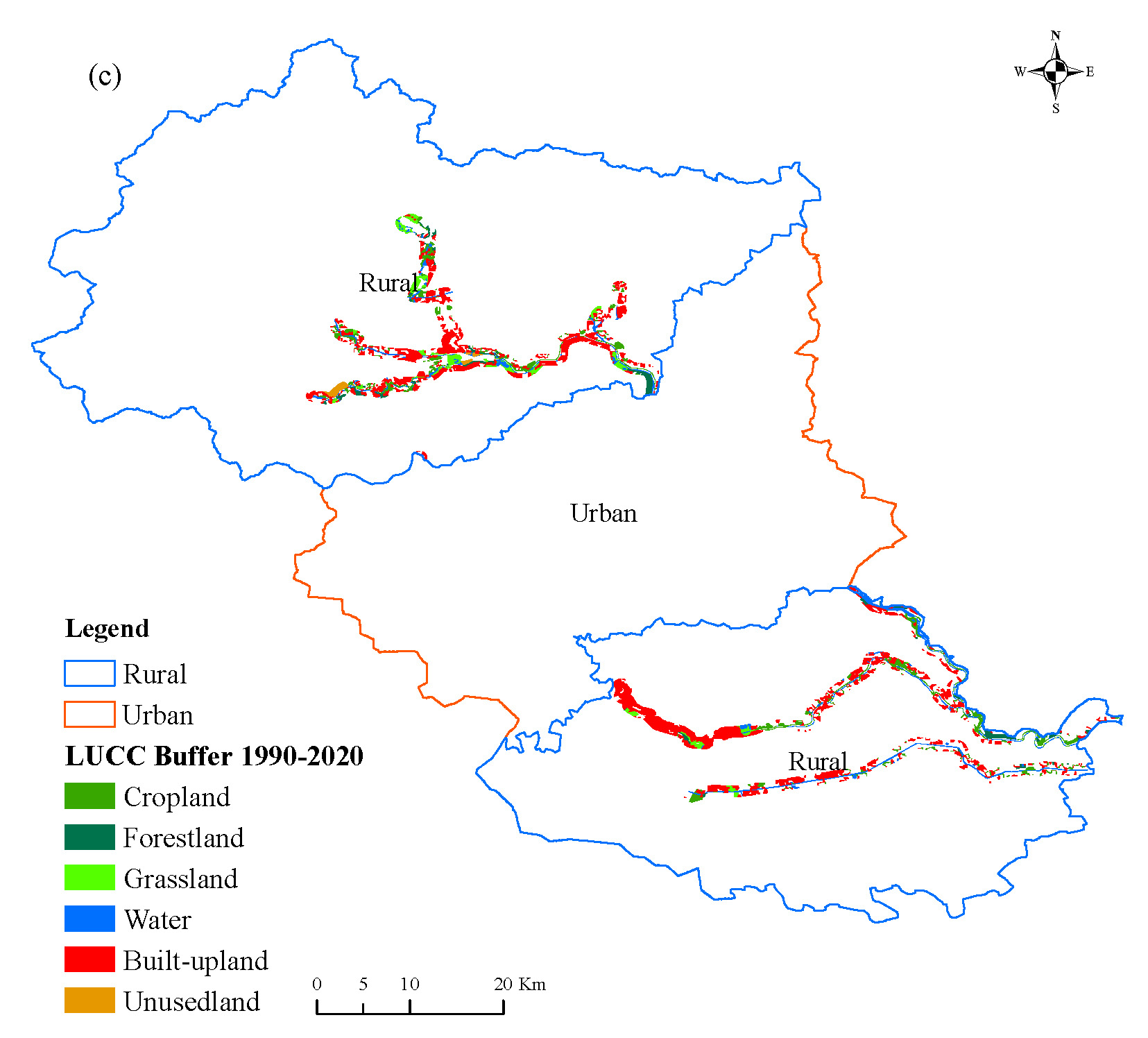


Fig. S2 Land-use transfer for rural corridors with width of 400m(a), 500m(b), 600m(c) from 1990 to 2020.

**Table S1 Landscape Pattern Indices of Rural and Urban Ecological Corridors from 1990 to 2020**

| Corridors | Width | LPI (%) | ED(m/ha) | LSI | CONTAG (%) | AI (%) | PAFRAC | PD (Patch/km^2^) | SHDI | SHEI |
| --- | --- | --- | --- | --- | --- | --- | --- | --- | --- | --- |
| Rural | 100 | 16.7±5.05 | 5.05±50.88 | 50.88±11.06 | 11.06±28.14 | 28.14±1.74 | 1.74±60.19 | 60.19±3.68 | 3.68±83.81 | 83.81±1.62 |
|  | 200 | 15.91±3.41 | 3.41±49.56 | 49.56±10.15 | 10.15±25.22 | 25.22±2.26 | 2.26±60.31 | 60.31±3.07 | 3.07±88.54 | 88.54±1.49 |
|  | 300 | 14.72±3.66 | 3.66±42.34 | 42.34±9.14 | 9.14±23.12 | 23.12±2.47 | 2.47±61.3 | 61.3±3.27 | 3.27±91.07 | 91.07±1.34 |
|  | 400 | 14.09±3.94 | 3.94±37.9 | 37.9±8.23 | 8.23±21.8 | 21.8±2.55 | 2.55±62.95 | 62.95±3.79 | 3.79±92.46 | 92.46±1.2 |
|  | 500 | 14.14±3.16 | 3.16±35.59 | 35.59±7.83 | 7.83±21.24 | 21.24±2.69 | 2.69±63.55 | 63.55±3.7 | 3.7±93.22 | 93.22±1.15 |
|  | 600 | 14.04±3.25 | 3.25±33.85 | 33.85±7.5 | 7.5±20.92 | 20.92±2.81 | 2.81±64.03 | 64.03±3.64 | 3.64±93.75 | 93.75±1.1 |
| Urban | 50 | 24.45±12.56 | 12.56±25.63 | 25.63±17.92 | 17.92±22.79 | 22.79±1.48 | 1.48±67.29 | 67.29±12.73 | 12.73±78.96 | 78.96±2.48 |
|  | 100 | 29.53±14.11 | 14.11±26.54 | 26.54±16.19 | 16.19±17.68 | 17.68±1.9 | 1.9±66.57 | 66.57±12.02 | 12.02±87.89 | 87.89±2.25 |
|  | 150 | 28.64±17.28 | 17.28±28.37 | 28.37±15.83 | 15.83±16.01 | 16.01±2.27 | 2.27±66.22 | 66.22±12.44 | 12.44±90.54 | 90.54±2.2 |
|  | 200 | 28.8±22.05 | 22.05±27.52 | 27.52±14.61 | 14.61±14.84 | 14.84±2.41 | 2.41±65.82 | 65.82±12.11 | 12.11±92.12 | 92.12±2.02 |
|  | 250 | 27.82±22.29 | 22.29±25.78 | 25.78±13.62 | 13.62±13.93 | 13.93±2.51 | 2.51±66.14 | 66.14±11.58 | 11.58±93.25 | 93.25±1.88 |
|  | 300 | 27.67±21.95 | 21.95±24.07 | 24.07±12.75 | 12.75±13.26 | 13.26±2.57 | 2.57±66.23 | 66.23±11.27 | 11.27±94.05 | 94.05±1.76 |

Note: The data in the table were presented as arithmetic mean ± standard deviation.

**Table S2 Sensitivity index of ecosystem service-function values and land uses in the North Canal River basin from 1990 to 2020.**

| LUCC | 1990 | | 1995 | | 2000 | | 2005 | | 2010 | | 2015 | | 2020 | |
| --- | --- | --- | --- | --- | --- | --- | --- | --- | --- | --- | --- | --- | --- | --- |
|  | Rural | Urban | Rural | Urban | Rural | Urban | Rural | Urban | Rural | Urban | Rural | Urban | Rural | Urban |
| Cropland | 0.21 | 0.22 | 0.16 | 0.12 | 0.16 | 0.11 | 0.15 | 0.09 | 0.19 | 0.08 | 0.17 | 0.07 | 0.13 | 0.05 |
| Grassland | 0.56 | 0.07 | 0.53 | 0.06 | 0.53 | 0.06 | 0.56 | 0.05 | 0.63 | 0.04 | 0.64 | 0.04 | 0.62 | 0.09 |
| Forestland | 0.01 | 0.00 | 0.01 | 0.00 | 0.01 | 0.00 | 0.01 | 0.00 | 0.02 | 0.01 | 0.01 | 0.01 | 0.03 | 0.04 |
| Water | 0.22 | 0.28 | 0.30 | 0.29 | 0.30 | 0.29 | 0.28 | 0.25 | 0.16 | 0.09 | 0.17 | 0.05 | 0.22 | 0.09 |
| Build-upland | 0.00 | 0.42 | 0.00 | 0.53 | 0.00 | 0.54 | 0.00 | 0.61 | 0.00 | 0.78 | 0.00 | 0.84 | 0.00 | 0.72 |
| Unused land | 0.00 | 0.00 | 0.00 | 0.00 | 0.00 | 0.00 | 0.00 | 0.00 | 0.00 | 0.00 | 0.00 | 0.00 | 0.00 | 0.00 |
